# Clinicoepidemiological and Dermoscopic Patterns of Hypopigmented Disorders: A Cross-Sectional Observational Study

**DOI:** 10.7759/cureus.110394

**Published:** 2026-06-07

**Authors:** Jithin Jayaraj, Yogesh Devaraj, Mukunda R Swaroop, Jagath Jayaraj, Shreya Rangaswamy, Jayaprakash MH, Akshatha V Reddy, Shifali Harinath

**Affiliations:** 1 Dermatology, Adichunchanagiri Institute of Medical Sciences, Adichunchanagiri University, Mandya, IND; 2 Dermatology, Akash Institute of Medical Sciences, Bangalore, IND; 3 Dermatology, Great Western Hospitals NHS Foundation Trust, Swindon, GBR

**Keywords:** dermoscopy, hypopigmented disorders, idiopathic guttate hypomelanosis, pityriasis versicolor, vitiligo

## Abstract

Introduction

Hypopigmented disorders comprise a heterogeneous group of dermatoses characterized by hypopigmented macules, patches, plaques, papules, and scaling lesions. These disorders often follow a chronic course and significantly affect patients' quality of life. Clinical differentiation may be challenging because of overlapping clinical presentations and atypical morphology. Dermoscopy serves as a non-invasive diagnostic tool that enhances visualization of submacroscopic morphological features and improves diagnostic accuracy.

Materials and methods

This cross-sectional observational study included 150 patients with hypopigmented disorders attending the dermatology outpatient department over a period of 18 months. The study population comprised 84 (56%) females and 66 (44%) males, with a mean age of 30.2 ± 2.57 years. Patients fulfilling the inclusion and exclusion criteria were enrolled after obtaining informed consent and institutional ethics committee's approval. Detailed history taking and clinical examination were performed using a predesigned proforma. Dermoscopic evaluation was conducted using polarized and non-polarized modes at 10× magnification. Clinical and dermoscopic findings were documented photographically and analyzed.

Results

Among 150 patients, pityriasis versicolor was the most common disorder, observed in 36 (24%) patients, followed by vitiligo in 33 (22%) patients and pityriasis alba in 30 (20%) patients. Reduced pigment network was the most consistent dermoscopic finding and was observed in all 150 (100%) cases. Scaling was predominantly observed in pityriasis versicolor in 25 (69.4%) patients and pityriasis alba in 18 (60%) patients. Vascular structures were commonly observed in lichen sclerosus in nine (90%) patients, while leukotrichia was characteristic of vitiligo and was seen in 13 (39.4%) patients. Additional findings such as pseudopod-like extensions, perifollicular pigmentation, comet-tail appearance, and double-edged scales assisted in differentiating specific disorders. Dermoscopic findings showed a strong correlation with clinical diagnosis.

Conclusion

Dermoscopy is a reliable and non-invasive adjunctive tool in the evaluation of hypopigmented disorders. In addition to a reduced pigment network, characteristic findings such as scaling patterns, vascular morphology, follicular changes, and border alterations improve diagnostic precision and help distinguish clinically similar conditions. Incorporation of dermoscopy into routine dermatological practice enhances diagnostic confidence and may reduce the need for invasive procedures.

## Introduction

Hypopigmentary skin conditions represent a broad and varied category of dermatological diseases, typically identified by whitish patches or macules caused by a loss or decrease in melanin [1,2]. Although these disorders are frequently encountered, establishing an accurate clinical diagnosis can be challenging. This is largely because most of the clinical entities often exhibit similar clinical features, particularly in atypical presentations or during the early stages of disease [1,2]. For example, disorders like vitiligo, pityriasis alba (PA), pityriasis versicolor (PV), idiopathic guttate hypomelanosis (IGH), lichen sclerosus et atrophicus (LSA), and nevus depigmentosus (ND) share many visual traits. Consequently, careful evaluation is required to establish the right treatment plan and prognosis [3].

For a long time, tissue biopsies for histopathological review have been the absolute gold standard for diagnosing these issues. However, the invasive nature of a biopsy makes it far from ideal for everyday clinic visits or for pediatric patients [4]. Recently, dermoscopy has emerged as a rapid, non-invasive diagnostic tool that connects what we see on the surface with what is happening at the tissue level [4,5]. By using high magnification combined with transillumination, dermoscopes reveal subtle structural details, like changes in the pigment network, blood vessel arrangements, scaling patterns, and hair follicle issues, that are not visible to the naked eye [4]. This technology is highly effective at distinguishing actual depigmentation from mere hypomelanosis and pinpointing clues unique to specific diseases [6].

While dermoscopy is widely recognized for assessing darker, pigmented lesions, there is surprisingly little research on its use for hypopigmentary conditions, especially in patients with darker skin tones (Fitzpatrick types III-V) [6,7]. Previous studies have reported characteristic dermoscopic findings in hypopigmentary disorders, including diffuse white glow in vitiligo, double-edged scales in pityriasis versicolor, pseudopod-like extensions in idiopathic guttate hypomelanosis, and perifollicular pigmentation in nevus depigmentosus, which have been shown to improve diagnostic accuracy [6,7]. Because skin of color has a naturally higher baseline of epidermal melanin, the visual contrast during dermoscopic examination is altered. This means that doctors need to learn diagnostic patterns specific to these demographics [6].

Therefore, the purpose of this study was to explore how clinical presentations correlate with their corresponding dermoscopic findings. By doing so, we aim to reduce misdiagnoses, improve targeted treatments, and potentially eliminate the need for invasive procedures like skin biopsies.

## Materials and methods

This cross-sectional observational study was conducted in the Department of Dermatology, Venereology, and Leprosy at Adichunchanagiri Hospital and Research Centre, a tertiary care center in Southern India, from April 2024 to September 2025. Ethical clearance was obtained from the Institutional Ethics Committee, and written informed consent was obtained from all participants or from the legal guardians of minors, with assent obtained from minors whenever appropriate.

Consecutive convenience sampling was used. All consecutive patients with hypopigmented disorders attending the dermatology outpatient department who fulfilled the inclusion and exclusion criteria during the study period were included in the study. A total of 150 patients were enrolled, comprising 84 (56%) females and 66 (44%) males, with a mean age of 30.2 ± 2.57 years.

Inclusion criteria comprised patients with hypopigmentary disorders who were willing to participate in the study, children below 18 years whose parents or guardians provided informed consent, and patients previously treated for hypopigmented disorders with a treatment-free period of at least one month. Patients were excluded if they were unwilling to participate, had received topical or systemic therapy within the preceding one month, had secondarily infected lesions, or had active viral, bacterial, or neoplastic skin diseases that could alter dermoscopic findings.

Data were collected using a structured proforma. Baseline demographic data such as age, sex, and occupation were recorded. Fitzpatrick skin phototype was assessed clinically for all participants using the Fitzpatrick skin type classification system based on the individual's tendency to burn and tan following sun exposure. Patients were categorized as Fitzpatrick skin types III, IV, or V. Clinical history included details regarding symptom onset, duration, disease progression, lesion distribution, presence of pruritus, excessive sweating, use of occlusive clothing, previous similar lesions, remissions, seasonal variation, family history, and systemic comorbidities.

A detailed clinical examination was performed to assess the morphology, color, distribution, borders, and extent of the hypopigmented lesions. Relevant bedside observations, such as the presence of Koebner phenomenon, were documented when indicated. Photographic documentation of lesions was carried out after obtaining informed consent.

Dermoscopic evaluation was performed using the ILLUCO IDS-1100 dermatoscope (ILLUCO Co., Ltd., Gunpo-si, South Korea) with 10× magnification. The ILLUCO IDS-1100 dermatoscope includes both polarized and non-polarized modes and features a 25 mm lens diameter, 32 light-emitting diodes (LEDs), three brightness levels, and compatibility with smartphone adaptors. It operates on a rechargeable lithium-ion battery with a continuous duty time of two hours and a charging time of three hours.

Dermoscopic examination was performed on recent, active lesions using minimal pressure. Observations included the presence of altered pigmentation or pigment network reduction, background color (diffuse white glow, porcelain white, whitish-brown), border characteristics (well-defined, ill-defined, or serrated), scale color and distribution (branny, fine, double-edged), vascular morphology (linear and blanching), and follicular findings (leukotrichia, comedo-like openings). Special morphological clues such as pseudopod-like extensions, perilesional hyperpigmentation, and chrysalis strands were also evaluated. These dermoscopic criteria were selected based on expert input and previous literature [4,6,7].

Statistical analysis

Data were entered into Microsoft Excel (Microsoft Corporation, Redmond, WA) and analyzed using SPSS version 25.0 (IBM Corp., Armonk, NY). Descriptive statistics were used to summarize the data. Continuous variables were expressed as mean ± standard deviation, while categorical variables were presented as frequencies and percentages.

## Results

Clinical findings

This cross-sectional observational study included 150 patients with hypopigmented disorders, comprising 84 (56%) females and 66 (44%) males, with a mean age of 30.2 ± 2.57 years. The majority of patients belonged to the 0-10 years age group (25%), followed by the 21-30 years age group (17%). The mean age of the study population was 30.2 ± 2.57 years. Females constituted 84 (56%) patients, while males accounted for 66 (44%), showing a slight female predominance. Students represented the largest occupational group (36%), followed by farmers (17.33%) and teachers (14%).

Pruritus was the most common presenting symptom and was observed in 35% of patients. Lesions were present since birth in 12% of cases. Associated comorbidities or significant past medical history were noted in 13% of patients, while seasonal exacerbation was reported in 13% of cases. Excessive sweating was observed in 33% of patients, and the use of occlusive clothing was reported by 55% of the study population. Previous topical treatment prior to presentation was noted in 44% of cases. A positive family history was present in 3% of patients.

Pityriasis versicolor (PV) was the most common condition, accounting for 36 (24%) cases, followed by vitiligo in 33 (22%) patients, pityriasis alba (PA) in 30 (20%), idiopathic guttate hypomelanosis (IGH) in 23 (15.33%), nevus depigmentosus (ND) in 18 (12%), and lichen sclerosus et atrophicus (LSA) in 10 (6.67%) patients. The distribution of hypopigmented disorders among the study population is summarized in Table 1.

**Table 1:** Distribution of hypopigmented disorders among patients.

Clinical diagnosis	Number of patients (n)	Percentage (%)
Pityriasis versicolor	36	24.0
Vitiligo	33	22.0
Pityriasis alba	30	20.0
Idiopathic guttate hypomelanosis	23	15.3
Nevus depigmentosus	18	12.0
Lichen sclerosus et atrophicus	10	6.7
Total	150	100

The thorax was the most commonly involved site (38%), followed by the face (23%), chest (12%), and limbs (11%). Multiple lesions were present in 67% of patients. Patches were the predominant lesion morphology, observed in 72% of cases, followed by macules in 21% and papules in 6%. Ill-defined borders were identified in 55% of lesions, whereas 45% demonstrated well-defined borders. Hyperpigmentation of the surrounding skin was observed in 67% of cases.

The majority of patients belonged to Fitzpatrick skin type IV (75%), followed by type V (14%) and type III (11%). Koebner’s phenomenon was observed in 12% of patients.

Dermoscopic findings

Dermoscopy was performed in all patients. Reduced pigment network was the most consistent finding and was observed in all lesions irrespective of diagnosis. Brown globules were identified in 40% of cases, while blue-gray dots were observed in 7% of patients. Vascular structures were absent in the majority of lesions (81.33%); however, linear blanching vessels were identified in 19% of cases.

Scaling was absent in 67% of patients. Among lesions demonstrating scales, branny scales were the most common finding (17%), followed by fine scales (12%). Double-edged scales were identified in 5% of patients and were predominantly associated with pityriasis versicolor.

Follicular findings included leukotrichia in 9% of cases and comedo-like structures in 7%. Perilesional hyperpigmentation and perifollicular hyperpigmentation were observed in 49% and 29% of patients, respectively. Pseudopod-like extensions were identified in 20% of cases.

**Figure 1:**
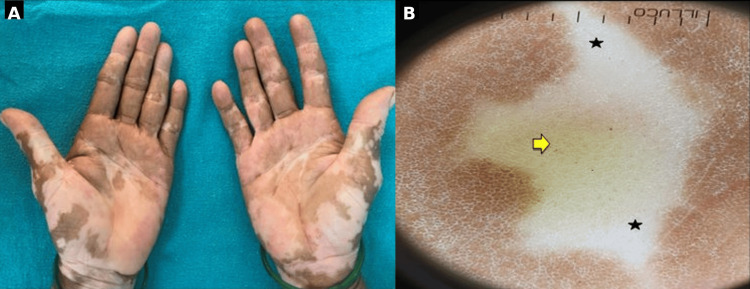
Clinical and dermoscopic images of vitiligo vulgaris (10× magnification). (A) Clinical image of vitiligo vulgaris showing multiple white patches on the bilateral palms. (B) Dermoscopic view in a well-established and stable stage of vitiligo showing diffuse white glow (black stars) and residual pigmentation (yellow arrow).

Vitiligo characteristically demonstrated diffuse white glow, leukotrichia, and perifollicular pigmentation (Figure 1).

**Figure 2:**
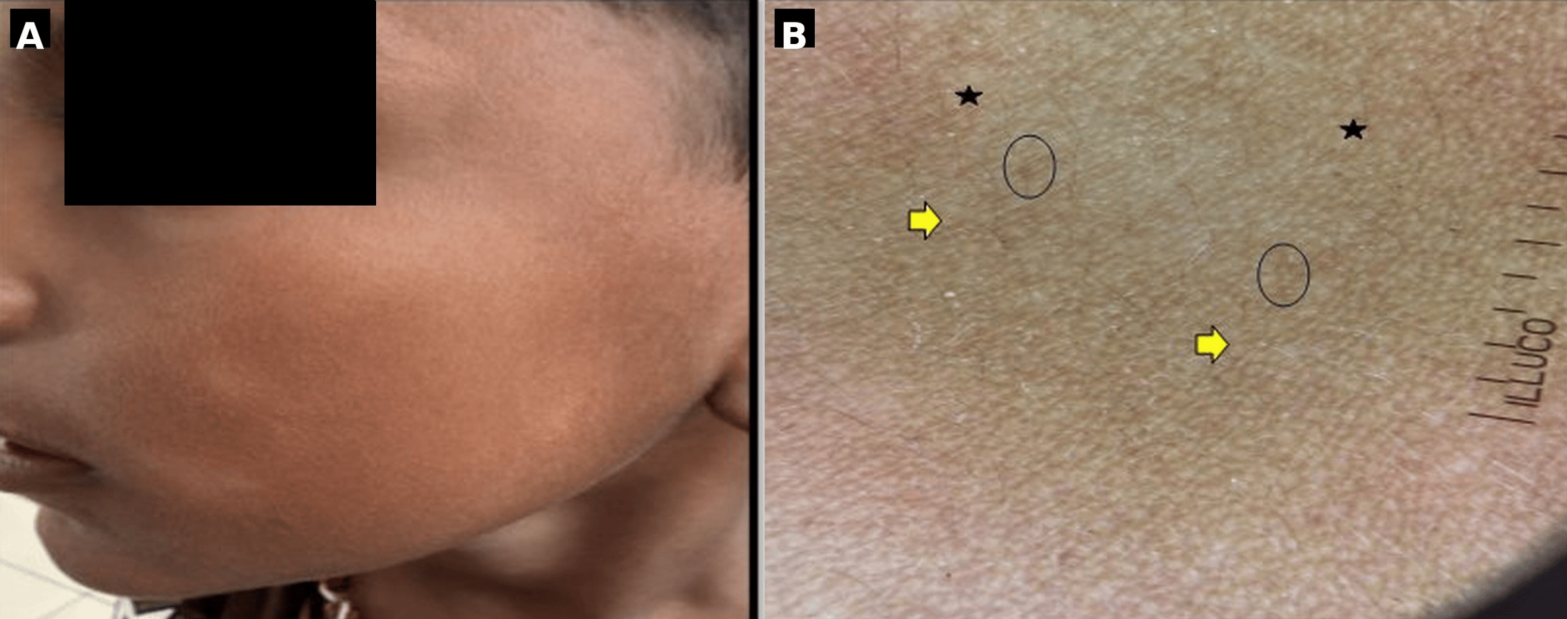
Clinical and dermoscopic images of pityriasis alba (10× magnification). (A) Clinical picture of pityriasis alba showing hypopigmented patches with fine scales on the left cheek. (B) Dermoscopic view of pityriasis alba showing white structureless areas (stars) with faint pigment network (circles) and white scales (yellow arrows).

Pityriasis alba predominantly showed ill-defined hypopigmented patches with fine scales (Figure 2).

**Figure 3:**
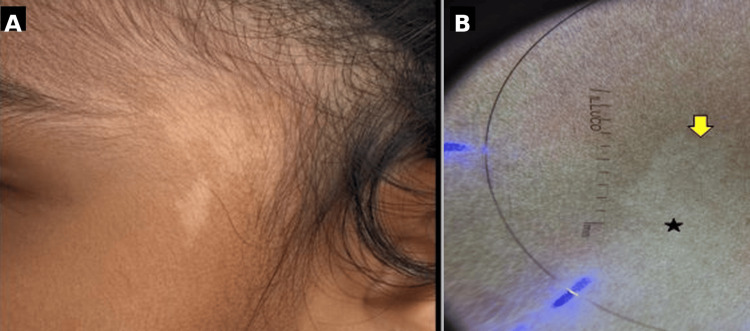
Clinical and dermoscopic images of nevus depigmentosus (10× magnification). (A) Clinical image of nevus depigmentosus showing a well-defined depigmented patch with serrated borders. (B) Dermoscopic view in nevus depigmentosus showing white areas (black star) with pseudopod-like extensions (arrows) at the periphery.

Nevus depigmentosus demonstrated serrated borders and perifollicular pigmentation (Figure 3).

**Figure 4:**
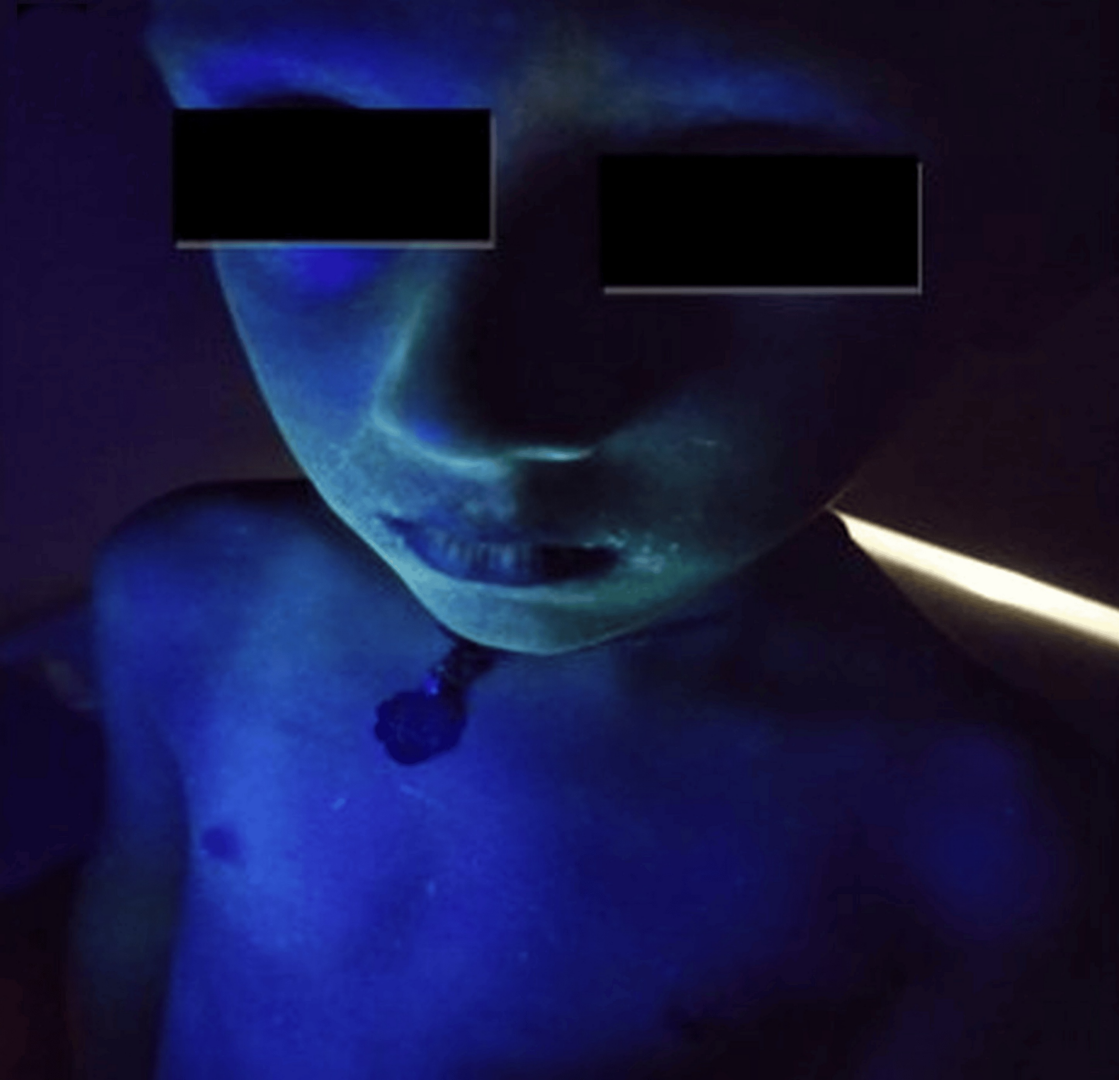
Wood’s lamp findings showing yellowish to yellow-green fluorescence in pityriasis versicolor.

**Figure 5:**
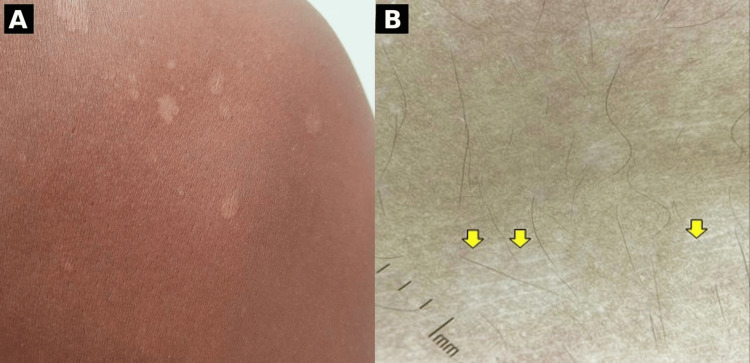
Clinical and dermoscopic images of pityriasis versicolor (10× magnification). (A) Clinical photograph of hypopigmented pityriasis versicolor showing well-defined scaly macules and patches on the shoulder. (B) Dermoscopic image of pityriasis versicolor showing prominent white scales in the skin lines (arrows) with a dull white background.

Pityriasis versicolor characteristically showed branny scales, comet-tail appearance, and double-edged scales, while Wood’s lamp examination demonstrated accentuation of lesions with yellowish fluorescence (Figures 4, 5).

**Figure 6:**
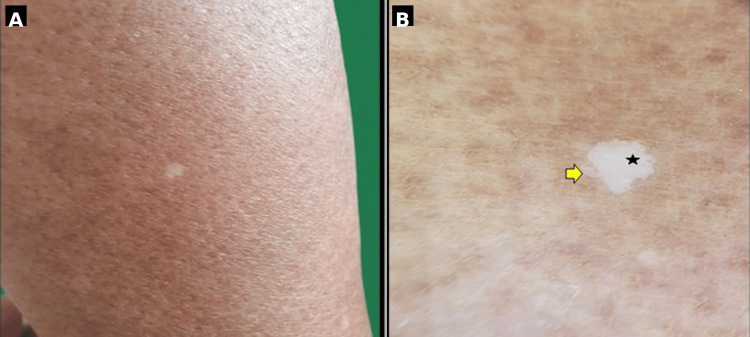
Clinical and dermoscopic image of idiopathic guttate hypomelanosis (10× magnification). (A) Clinical image of idiopathic guttate hypomelanosis showing a single depigmented macule on the forearm. (B) Dermoscopy in an amoeboid pattern of idiopathic guttate hypomelanosis reveals white structureless areas (black stars) that extend peripherally like pseudopods (arrows) of an amoeba.

Idiopathic guttate hypomelanosis characteristically demonstrated porcelain-white macules with pseudopod-like extensions (Figure 6).

**Figure 7:**
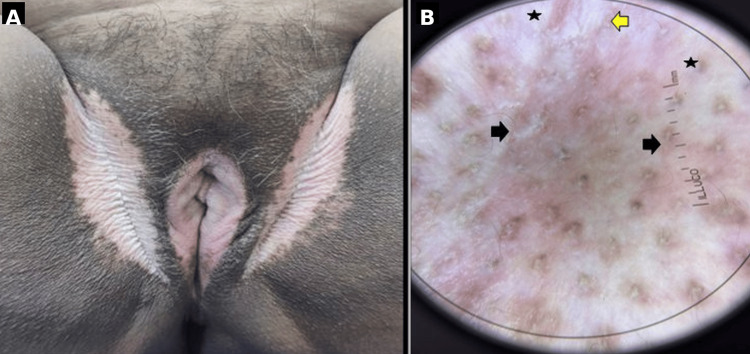
Clinical and dermoscopic images of lichen sclerosus et atrophicus (10× magnification). (A) Clinical image of lichen sclerosus et atrophicus on the female genital region showing well-defined whitish plaques. (B) Dermoscopic view of the inflammatory stage of lichen sclerosus et atrophicus showing comedo-like openings (black arrows), white areas (black stars), and linear vessels (yellow arrow).

Lichen sclerosus et atrophicus showed chrysalis-like structures, blue-gray dots, and linear vessels on dermoscopy (Figure 7).

Among the special dermoscopic clues observed, serrated borders were the most common finding, present in 18 (12%) patients, followed by chrysalis strands in 10 (6.67%) and cloudy sky appearance in seven (4.67%) patients. Comet-tail appearance and starburst appearance were observed in five (3.33%) patients each. Porcelain-white macules were identified in four (2.67%) cases, while feather-like spreading margins were noted in three (2%) patients. Special dermoscopic clues were absent in 92 (61.33%) cases. The distribution of special dermoscopic clues observed among patients is presented in Table 2.

**Table 2:** Distribution of special dermoscopic clues among patients.

Special dermoscopic clue	Number of patients (n)	Percentage (%)
Serrated borders of patches	18	12.0
Chrysalis strands	10	6.7
Cloudy sky appearance	7	4.7
Comet-tail appearance	5	3.3
Starburst appearance	5	3.3
Porcelain white macule	4	2.7
Feather-like spreading margins	3	2.0
Absent	92	61.3
Total	150	100

Distinct associations between dermoscopic findings and specific hypopigmented disorders were identified. The association between dermoscopic patterns and specific hypopigmented disorders is summarized in Table 3.

**Table 3:** Association of dermoscopic patterns with hypopigmented disorders. IGH: idiopathic guttate hypomelanosis; LSA: lichen sclerosus et atrophicus; ND: nevus depigmentosus; PA: pityriasis alba; PV: pityriasis versicolor.

Dermoscopic feature	IGH (n = 23)	LSA (n = 10)	ND (n = 18)	PA (n = 30)	PV (n = 36)	Vitiligo (n = 33)
Background color	Porcelain white in 23 (100%)	Pinkish white in 10 (100%)	Dull white in 5 (28.7%)	Whitish brown in 30 (100%)	Whitish brown in 36 (100%)	Diffuse white glow in 26 (79%)
Well-defined borders	19 (82.6%)	10 (100%)	18 (100%) (serrated in most cases)	0 (0%)	22 (61.1%)	16 (48.5%)
Ill-defined borders	4 (17.3%)	0 (0%)	0 (0%)	30 (100%)	14 (38.9%)	17 (51.5%)
Symmetrical lesions	15 (65.2%)	10 (100%)	0 (0%)	5 (16.7%)	16 (44.4%)	2 (6.1%)
Reduced pigment network	23 (100%)	10 (100%)	18 (100%)	30 (100%)	36 (100%)	33 (100%)
Brown dots	2 (8.6%)	Blue-gray dots in 10 (100%)	0 (0%)	18 (60%)	9 (25%)	12 (36.4%)
Brown globules	11 (47.8%)	Blue-gray globules in 10 (100%)	0 (0%)	11 (36.7%)	24 (66.7%)	14 (42.4%)
Follicular findings	Absent in 23 (100%)	Comedo-like openings in 10 (100%)	Perifollicular pigmentation in 18 (100%)	Comedo-like openings in 30 (100%)	Comedo-like openings in 36 (100%)	Leukotrichia in 13 (39.4%)
Vascular structures	Absent	Linear blanching vessels in 9 (90%)	Absent	Absent	Absent	Absent
Scales present	0 (0%)	0 (0%)	0 (0%)	Fine scales in 18 (60%)	Branny scales in 25 (69.4%); diffuse white scales in 4 (11.1%)	0 (0%)
Perilesional hyperpigmentation	12 (50%)	0 (0%)	18 (100%)	5 (16.7%)	12 (33.3%)	26 (78.8%)
Perifollicular pigmentation	0 (0%)	0 (0%)	18 (100%)	0 (0%)	6 (15.6%)	14 (42.4%)
Double-edged scales	0 (0%)	0 (0%)	0 (0%)	0 (0%)	7 (19.4%)	0 (0%)
Pseudopod-like extensions	13 (56.5%)	0 (0%)	17 (94.4%)	0 (0%)	0 (0%)	0 (0%)
Special dermoscopic clues	Amoeboid in 14 (60.9%), feathery in 6 (26.1%), petaloid in 2 (8.7%), nebuloid in 1 (4.3%)	Chrysalis-like structures in 6 (60%)	Pseudopod extensions in 17 (94.4%)	No specific clue	Comet-tail appearance in 5 (15.1%), sago-grain appearance in 12 (33.3%), starburst appearance in 4 (12.1%)	White glow and leukotrichia in 13 (39.4%)

A porcelain-white background with pseudopod-like extensions was predominantly associated with IGH. LSA characteristically demonstrated chrysalis-like structures and linear blanching vessels. ND consistently showed serrated borders with perifollicular pigmentation. Fine scales and ill-defined borders were characteristic findings in pityriasis alba, whereas branny scales and double-edged scales were predominantly associated with pityriasis versicolor. Vitiligo demonstrated diffuse white glow, leukotrichia, and perifollicular pigmentation as major dermoscopic findings.

## Discussion

Clinical findings

In the present study of 150 patients with hypopigmented disorders, the majority of patients were within the first decade of life (25%), followed by the 21-30 years age group (17%), with a mean age of 30.2 ± 2.57 years. Females constituted 56% of the study population, with a female-to-male ratio of 1.27:1. Similar demographic trends with female preponderance and early onset of hypopigmented dermatoses were reported by Al-Refu [8] and Mareddy et al. [2]. Pruritus was the predominant symptom, observed in 35% of patients, comparable to findings reported by Al-Refu [8]. Excessive sweating and the use of occlusive clothing were noted in 33% and 55% of patients, respectively, predominantly among cases of pityriasis versicolor. Similar associations have been reported in previous studies of pityriasis versicolor [9]. Seasonal exacerbation was observed in 13.3% of patients, while prior topical medication use was reported in 44% of cases.

Koebner’s phenomenon was noted in 12% of patients, particularly in vitiligo and lichen sclerosus et atrophicus, consistent with its recognized association with disease activity in vitiligo [10].

Students formed the largest occupational group (36%), reflecting the younger age distribution observed in the study population [11]. Similar observations have also been reported in pediatric populations with hypopigmented disorders [12].

This observation may be attributed to the known role of warm, humid conditions, increased perspiration, and occlusion in promoting the proliferation of Malassezia species, which are implicated in the pathogenesis of pityriasis versicolor [13].

A positive family history was observed in 3% of patients, whereas lesions were present since birth in 12% of cases, predominantly in nevus depigmentosus. Similar congenital onset patterns have been described by Patel et al. [3] in localized functional hypomelanosis.

In the present study, pityriasis versicolor was the most common hypopigmented disorder (24%), followed by vitiligo (22%), pityriasis alba (20%), idiopathic guttate hypomelanosis (15%), nevus depigmentosus (12%), and lichen sclerosus et atrophicus (7%). Similar distributions have been reported in previous clinicoepidemiological studies of hypopigmented disorders [2,11].

Associated comorbidities or significant past medical history were noted in 13.33% of patients. However, the specific comorbid conditions were not analyzed separately in the present study. Although lower than the prevalence reported in broader epidemiological studies by Mareddy et al. [2], this finding remains clinically significant, particularly in disorders with known systemic associations such as vitiligo.

Patches were the most common lesion morphology, accounting for 72% of lesions, followed by macules in 21% of cases [11,12]. Ill-defined borders were observed in 55% of patients, predominantly in pityriasis alba and pityriasis versicolor. Perilesional hyperpigmentation was present in 67% of cases.

Dermoscopic findings

A reduced pigment network was observed universally across all hypopigmented lesions in the present study. Similar findings were described by Errichetti and Stinco [4] and Al-Refu [8], who identified a reduced or absent pigment network as a characteristic dermoscopic hallmark of hypomelanotic disorders. Brown globules were observed in 40% of cases, while blue-gray peppering was identified in 6.67%.

Vitiligo

Vitiligo demonstrated a characteristic diffuse white glow on dermoscopy, correlating closely with observations by Kumar Jha et al. [10]. Similar peripheral pigmentary patterns have also been described in idiopathic guttate hypomelanosis [14]. Leukotrichia was observed predominantly in vitiligo lesions and represented a marker of melanocyte reservoir depletion [15]. The diffuse white glow reflects marked reduction or absence of epidermal melanin, while leukotrichia indicates loss of follicular melanocytes, which serve as an important reservoir for repigmentation [10,15]. Perilesional hyperpigmentation was noted in 49% of cases and may represent ongoing disease activity. Additional findings such as comet-tail appearance, sago-grain appearance, and starburst patterns were associated with evolving lesions.

Pityriasis Versicolor and Pityriasis Alba

Branny scaling was a prominent dermoscopic finding in pityriasis versicolor and was observed in 17% of the study population. Double-edged scales, considered highly specific for pityriasis versicolor [16], were identified in 5% of cases. Similar findings were reported by Kaur et al. [9], who observed scaling in 25 of 30 patients (83.33%), predominantly along the dermatoglyphics. The scaling observed in pityriasis versicolor corresponds to fungal colonization of the stratum corneum and the associated disruption of normal keratinization [9,13,16].

Pityriasis alba consistently demonstrated ill-defined borders with focal fine scaling. Similar findings were documented by Al-Refu [8], who reported ill-defined margins in most cases of pityriasis alba. Vascular structures were notably absent in the present study, supporting the chronic low-grade inflammatory nature of the condition. The ill-defined borders and fine scaling in pityriasis alba are attributed to mild epidermal inflammation and associated post-inflammatory hypomelanosis [8].

Idiopathic Guttate Hypomelanosis and Nevus Depigmentosus

Idiopathic guttate hypomelanosis and nevus depigmentosus exhibited characteristic marginal dermoscopic patterns. Pseudopod-like extensions were observed in 20% of cases, predominantly among patients with idiopathic guttate hypomelanosis and nevus depigmentosus. These marginal patterns likely correspond to the irregular distribution of residual melanocytes and melanin at the lesional margins [6,14]. Serrated borders were predominantly associated with nevus depigmentosus and likely reflect an inherent defect in melanosome transfer.

Lichen Sclerosus Et Atrophicus

Lichen sclerosus et atrophicus demonstrated prominent vascular and follicular alterations on dermoscopy. Linear and blanching vessels were observed along with comedo-like structures, correlating with the findings of Al-Refu [8], who identified vascular changes and follicular plugging as important dermoscopic clues. These findings correspond histologically to epidermal atrophy, follicular plugging, dermal sclerosis, and superficial vascular dilatation [8].

Overall, dermoscopy demonstrated significant correlation with clinical diagnosis and proved valuable in differentiating various hypopigmented disorders, consistent with findings from published literature [17,18]. The present study highlights the role of dermoscopy as a rapid, non-invasive diagnostic modality that bridges clinical morphology and histopathology, thereby improving diagnostic accuracy and potentially reducing the need for invasive procedures [19].

Limitations

This study had a few limitations. As it was conducted in a single tertiary care hospital, the findings may not reflect the complete spectrum of dermoscopic patterns seen in other populations. Some disorders had comparatively fewer cases, which may have limited detailed comparison of certain dermoscopic features.

Since the study was cross-sectional, changes in lesions and dermoscopic findings over time could not be evaluated. Dermoscopic interpretation may also vary between observers despite the use of standard criteria. Most patients belonged to Fitzpatrick skin type IV, which may reduce the applicability of the findings to other skin types. In addition, histopathological confirmation was not available in all cases because the diagnosis was mainly based on clinical and dermoscopic assessment. Further multicentric studies with larger sample sizes and histopathological correlation would help strengthen these observations.

## Conclusions

Dermoscopy is a highly valuable, non-invasive diagnostic tool that significantly enhances the clinical evaluation of hypopigmented disorders in skin of color. While a reduced pigment network represents a universal finding across all such conditions, disorder-specific parameters, such as the double-edged scales in pityriasis versicolor, pseudopod extensions in idiopathic guttate hypomelanosis, serrated borders in nevus depigmentosus, and prominent vascularity in lichen sclerosus, allow for reliable differentiation.

Integrating dermoscopy into routine dermatological practice improves diagnostic precision, facilitates earlier targeted interventions, and reduces the reliance on invasive skin biopsies.
